# Supplementary material for: Polygenic risk score for Alzheimer’s disease and trajectories of cardiometabolic risk factors in children
Source: Wellcome Open Res. 2019 Aug 20;4:125. [Version 1] doi: 10.12688/wellcomeopenres.15359.1 (PMC6971849; doi:10.12688/wellcomeopenres.15359.1)
Supplement: Peer review history

# Peer review: Reviewer response for version 1

**DOI:** 10.21956/wellcomeopenres.16777.r37376

**Published:** 2020-01-20

**Authors:** Michelle Grunin

**Affiliations:** 1 Department of Population and Quantitative Health Sciences, Case Western Reserve University, Cleveland, OH, USA

Korologou-Linden *et al.*'s work aims to investigate whether polygenic risk scores (PRS) for Alzheimer’s disease are associated with cardiometabolic measures and findings throughout the longitudinal Avon Longitudinal Study of Parents and children. They analyzed between 699 and 7,977 participants depending on the measure used, to determine if these factors throughout childhood and adolescences are associated with the PRS for Alzheimer’s Disease.

 The paper’s statistics were very well done, including multiple different methodology depending on the factors and analyses. The results were null, but extremely interesting, and indexing this would contribute to the knowledge in the field.

 A few minor comments:

1. The PRS were taken from the GWAS performed by the IGAP consortium, from their article from 2013. However IGAP has since published an updated meta-analysis ( Kunkle *et al.* 2019), and some of the SNPs mentioned may not have been included in the PRS proposed in this paper. If some significant SNPs were not included, perhaps the authors could mention if this would affect the null results, despite the fact that most of the previous LOAD associated SNPs originally found in 2013 were again found in 2019 (only 5 new GWAS loci were found in 2019). In addition, the expanded and more liberal thresholds of the PRS would also change if expanded further to the 2019 article.
2. APOE was excluded from most of the analysis as the effect would be confounded by its association with cardiometabolic issues. However, why was sensitivity analyses performed including APOE if so?
3. Why do the authors think that the direction of the effect differed between GWAS thresholds and more liberal thresholds for some risk factors (Supplementary Table 7)?

I confirm that I have read this submission and believe that I have an appropriate level of expertise to confirm that it is of an acceptable scientific standard.

# Peer review: Reviewer response for version 1

**DOI:** 10.21956/wellcomeopenres.16777.r36271

**Published:** 2019-09-24

**Authors:** Valentina Escott-Price, Emily Baker

**Affiliations:** 1 Dementia Research Institute at Cardiff, Cardiff University, Cardiff, UK; 2 Medical Research Council Centre for Neuropsychiatric Genetics and Genomics, Division of Psychological Medicine and Clinical Neurosciences, Cardiff University, Cardiff, UK

Korologou-Linden *et al*. aim to investigate whether polygenic risk scores for Alzheimer’s disease were associated with cardiometabolic health indicators through childhood and adolescence in a sample of 7,977 participants from the Avon Longitudinal Study of Parents and Children. The results are null, however, we support the authors in wishing to publish these results. The paper is well written but requires some clarification. 

 In the results section of the abstract, the authors state that no associations were observed excluding APOE gene region. Do they see association with APOE gene? Please mention this in the abstract.

 Genetic Data: How many SNPs in the data? How was 1 child per family selected? Randomly?

 In the Statistical analysis section the authors say that they excluded APOE from the PRS (gene or region?). Do they again excluded APOE in the sensitivity analyses section? The sentence “… *the PRS excluding ApoE and cardiometabolic risk factors at different p-value threshold*” is not clear. Is it SNP association p-values or cardiometabolic risk factors p-values? Could the authors please clarify in the statistical analyses a) exact positions of APOE region which was excluded; b) why the APOE tagging SNPs where included in the sensitivity analyses; c) clarify what other models were used for the sensitivity analyses and why .

 In the Results section, the sentences “ *Sensitivity analyses comparing the results for the PRS at different p-value thresholds used PRS excluding ApoE. Further analyses comparing the results with the PRS including/excluding ApoE were performed using the genome-wide significant threshold*” are not clear. According to the methods section “the exclusion of APOE” model is a part of the main analysis. In addition, why were “including apoe” models only tested for GWAS significant SNPs in the PRS? 

 Generating polygenic risk scores: How was APOE excluded? Just the APOE SNPs, or the whole APOE locus? How was linkage disequilibrium between SNPs handled? If LD pruning was used, which parameters were used, i.e. r2 threshold.

 Figures - the figures seem slightly blurred, a higher resolution would be nice. Perhaps Figures 1 & 3 could go to supplemental material?

 Was the correlation between HDL, non-HDL, CRP and triglyceride levels in a consistent direction at all time points?

 In the discussion section, it may also be that the effect sizes are very small and the sample size is not sufficient to reach the statistical significance (N ranged between ~700 and 7,000).

 Minor points:

 The following text should be reworded:

- Abstract, method: ‘birth at 18 years’.
- Pg. 3 Intro paragraph 2 ‘can be summarised to construct a polygenic risk score’.
- Pg. 3 Methods paragraph 1, keep numbers consistent ‘seven, nine, 10, …’ There are other instances of this through the paper.
- Pg. 8 strengths and limitations ‘in early in the life course’.

We confirm that we have read this submission and believe that we have an appropriate level of expertise to confirm that it is of an acceptable scientific standard.
